# Designing a HER2/*neu *promoter to drive α1,3galactosyltransferase expression for targeted anti-αGal antibody-mediated tumor cell killing

**DOI:** 10.1186/bcr1034

**Published:** 2005-05-03

**Authors:** Marion Lanteri, Laurence Ollier, Valérie Giordanengo, Jean-Claude Lefebvre

**Affiliations:** 1 INSERM U526, Laboratoire de Virologie, Faculté de Médecine, avenue de Valombrose, 06107, Nice cedex 2, France

## Abstract

**Introduction:**

Our goal was to specifically render tumor cells susceptible to natural cytolytic anti-αGal antibodies by using a murine α1,3galactosyltransferase (mαGalT) transgene driven by a designed form of HER2/*neu *promoter (p*Neu*), the transcription of which is frequently observed to be above basal in breast tumors. Indeed, the αGalT activity that promotes Galα1,3Galβ1,4GlcNAc-R (αGal) epitope expression has been mutationally disrupted during the course of evolution, starting from Old World primates, and this has led to the counter-production of large amounts of cytotoxic anti-αGal antibodies in recent primates, including man.

**Method:**

Expression of the endogenous *c-erbB-2 *gene was investigated in various cell lines by northern blotting. A mαGalT cDNA was constructed into pcDNA3 vector downstream of the original CMV promoter (pCMV/mαGalT) and various forms of p*Neu *were prepared by PCR amplification and inserted in the pCMV/mαGalT construct upstream of the mαGalT cDNA, in the place of the CMV promoter. These constructs were transferred into HEK-293 control and breast tumor cell lines. Stably transfected cells were analyzed by northern blotting for their expression of αGalT and c-erbB-2, and by flow cytometry for their binding with fluorescein isothiocyanate-conjugated *Griffonia simplicifolia*/isolectin B4.

**Results:**

We show that expression of the mαGalT was up- or down-modulated according to the level of endogenous p*Neu *activity and the particular form of constructed p*Neu*. Among several constructs, two particular forms of the promoter, p*Neu*250 containing the CCAAT box and the PEA3 motif adjacent to the TATAA box, and p*Neu*664, which has three additional PEA3 motifs upstream of the CCAAT box, were found to promote differential αGalT expression.

**Conclusion:**

Our results strengthen current concepts about the crucial role played by the proximal PEA3 motif of p*Neu*, and may represent a novel therapeutic approach for the development of targeted transgene expression.

## Introduction

The enzyme α1,3galactosyltransferase (αGalT) is responsible for the synthesis of galactose-α1,3galactose-β1,4N-acetylglucosamine-R (αGal) epitopes in all mammals except Old World primates [1]. Highly expressed in nonprimate mammals, prosimians and New World monkeys, this glycosyltransferase has been mutationally inactivated during the course of evolution, starting from Old World primates [2]. We have previously shown that, in human cells, transcription of the α*GalT *gene is interrupted by the presence of a strong stop signal in exon 7, which leads to a chimeric mRNA comprising the first four coding exons and part of intron VII, but lacking the last two exons corresponding to the catalytic domain of the enzyme [3]. As a consequence, and given the broad circulation of αGal carbohydrate antigens, humans, apes and Old World monkeys produce large amounts of anti-αGal antibodies, which represent approximately 1% of total IgG in humans [4]. These antibodies are responsible for the hyperacute rejection of xenografts and thus prevent trials on transplantation of pig organs to humans [5,6]. Conversely, they represent a potential constitutive tool for therapeutic applications because their highly efficient cytolytic activity could be directed against pathological cells transgenically modified to express αGal epitopes [7-10].

Gene therapy based on the induction of cytotoxicity generally makes use of transgenes that encode prodrug activating enzymes [11]. In the case of anti-αGal-induced cytotoxicity, no chemical drug is needed to obtain the effect of αGalT because natural circulating anti-αGal antibodies are sufficient to promote cell lysis via complement activation. One common problem with gene therapy is target cell selectivity. A frequent solution to this is the use of tissue-specific or tumor-activated promoters to drive expression of the transgene [12,13]. Human c-erbB-2 (synonyms erbB2, HER2/*neu*), a member of the *erbB *family that is overexpressed in about one third of breast tumors and in a variety of other tumors, is often correlated with a poor prognosis [14-16]. This gene is normally expressed at a low level in a variety of human embryonic and adult epithelial and hematopoietic cells [17,18]. The high overexpression of c-erbB-2 in tumor cells [19] results from multiple mechanisms, including gene amplification and transcription modulation [20-22]. c-erbB-2 is a 185 kDa transmembrane tyrosine kinase receptor related to the epidermal growth factor receptor that functions as a growth factor receptor to regulate cell growth and transformation [23-25]. Regulation of the *c-erbB-2 *promoter (p*Neu*) has been extensively investigated in a domain located within the 700 bp region upstream of its transcription start site. A -213/-87 fragment relative to the gene's transcription start site contains the minimal promoter region able to drive preferential transgene expression in breast cancer cells [26].

The present study was designed to obtain targeted expression of αGal epitopes by human breast cancer cells in order to render them susceptible to anti-αGal antibody-induced cell death. For this purpose, we used a murine αGalT (mαGalT) transgene driven by a form of p*Neu *designed to be significantly activated in breast tumor cells.

## Materials and methods

### Cells and reagents

The cell line HEK-293 (ATCC CRL-1573) and the human breast cancer cell lines MCF-7 (ATCC HTB-22), SK-BR-3 (ATCC HTB-30), MDA-MB-231 (ATCC HTB-26), and MDA-MB-453 (ATCC HTB-131) were cultured in Dulbecco's modified Eagle's medium (Gibco, Invitrogen, Rockeville, MD, USA) supplemented with 10% fetal calf serum (FCS; BioWhittaker, Rockland, ME, USA). Fluorescein isothiocyanate-conjugated *Griffonia simplicifolia */ isolectin B4 (FITC-GS-I-B4), which recognizes a terminal galactosyl residue in an α linkage, was purchased from EY Laboratories (San Matteo, CA, USA). The rabbit complement ORAX 07 was from Dade Behring (Paris, France).

### Murine αGalT constructions in a plasmid vector and transfection

The mαGalT cDNA was kindly provided by Uri Galili and cloned within *Hin*dIII/*Xba*I sites into pcDNA3 vector (Invitrogen, Cergy-Pontoise, France), downstream of the CMV early promoter (pCMV) or various truncated forms of the HER2/*neu *promoter (p*Neu*) obtained by PCR on genomic DNA extracted from human CEM cells (ATCC CCL-119) using the following primer sets: 5'-GGGGGTCCTGGAAGCCACAAG-3' and 5'-GTGCTCACTGCGGCTCCGGCC-3' for p*Neu*664 (-488/+176); 5'-TCGCGAGCAGGCAACCCAGGCGTCCCG-3' and 5'-AAGCTTCTCCCCTGGTTTCTCCGGTCCCAA-3' for p*Neu*250 (-216/+34); 5'-TCGCGAGCAGGCAACCCAGGCGTCCCG-3' and 5'-CCAAAAAGCTTGTGCTCACTGCGGCTCCGGCC-3' for p*Neu*392 (-216/+176); 5'-GGAAATCGCGAAGGAAGTATAAGAATGAAG-3' and 5'-CCAAAAAGCTTGTGCTCACTGCGGCTCCGGCC-3' for p*Neu*209 (-33/+176). All p*Neu *derivative forms were constructed within *Nru*I/*Hin*dIII sites upstream of mGalT cDNA in the place of pCMV.

Cells were transfected in six-well plates using FuGENE 6 transfection reagent (Roche Diagnostics, Meylan, France), as recommended by the manufacturer. Stably transfected cells were selected by G418 resistance.

### Murine αGalT constructions in a retroviral vector

To overcome the poor efficiency of classic methods of transfection in MDA-MB-231 cells, a retroviral vector system was developed. The undesirable promoting activity of the 5' long terminal repeat (LTR) was avoided by constructing the cassette p*Neu*250/mαGalT in a self-inactivating murine retroviral vector (pcPMΔU3) that had been prepared by removing nearly the entire U3 region of the 3' LTR (Lefebvre JC and March D, manuscript in preparation). Making use of this strategy, described in [27], the U3 deletion is transferred to the 5' LTR during the first retrotranscription of the retroviral construct, and further results in the transcriptional inactivation of the provirus in the infected cells. In addition, the cassette p*Neu*250/mαGalT was oriented in the opposite direction (3' to 5') to the LTR so as to completely rule out any residual viral promoter activity. To obtain retroviral particles pseudotyped with a vesicular stomatitis viral G glycoprotein (VSV-G), the plasmid construct was co-transfected in GP2-293 packaging cells with a pVSV-G vector (both from Clontech, BD Biosciences, Le Pont de Claix, France). Supernatants were harvested 48 h post-transfection and filtered (membrane pore size = 0.45 μm). VSV-G pseudotyped particles were concentrated by ultracentrifugation. Infected cells were seeded in 24-well plates (BD Falcon, Le Pont de Claix, France) and stably transduced subclones were selected by antibiotic resistance. Expression of m αGalT was evaluated using GS-I-B4 reactivity.

### Flow cytometry analysis

Phenotypic analyses were performed using FITC-GS-I-B4, as previously described [28]. Stained cells were analyzed on a FACScan cytometer (Becton Dickinson, San Jose, CA, USA).

### Northern blot analysis

Total RNAs were isolated using RNA Now reagent (Biogentex Inc., Seabrook, TX, USA), according to the manufacturer's instructions, and poly(A)-rich RNAs were selected as described elsewhere [29]. Poly(A)-rich RNAs (3 μg) were electrophoresed on denaturing 1.2% agarose gel and transfered in 20 × NaCl/Citrate onto a Hybond-N+ nylon membrane (Amersham-Biosciences, Saclay, France). Membranes were probed overnight at 42°C with [α-^32^P]-random-labeled mαGalT or *c-erbB-2 *cDNA and washed according to standard procedures. The mαGalT probe was excised from the pcDNA3 construct and the c-erbB-2 probe was obtained by PCR amplification on genomic DNA from SK-BR-3 cells with the primer set 5'-CCAGGAGGTGCAGGGCTACG-3' and 5'-ATCCTCAGAACTCTCTCCCC-3'. Membranes were exposed to Kodak XAR film (Eastman Kodak, Rochester, NY, USA). Detection of glyceraldehyde 3-phosphate-dehydrogenase (GAPDH) was used as an internal control.

### Cytotoxicity assay

Parental and transfected HEK-293, MCF-7, or SK-BR-3 cells were distributed in 96-well plates (5.10^4 ^cells/well) and incubated for 1 h after addition of 10 μl of various human sera, in triplicate. Rabbit complement (20 μl) was then added and plates were incubated for 1 h. Cell death was evaluated by the trypan blue vital dye exclusion method. The percentage of killed cells was evaluated by comparison of the number of blue-stained cells in the reaction and control wells.

### Cell proliferation assay

After cell incubation with human sera and rabbit complement as above, XTT reagent from the Cell Proliferation Kit II (Roche Diagnostics, Meylan, France) was added, according to the manufacturer's instructions, and the cells were re-incubated at 37°C for 2 h. Formazan formation was measured at 490 nm.

### Statistical analysis

Statistical comparison of mean values was performed with a one-way analysis of variance (ANOVA).

## Results

### Expression of the *c-erbB-2 *gene in various cell lines

**Figure 1:**
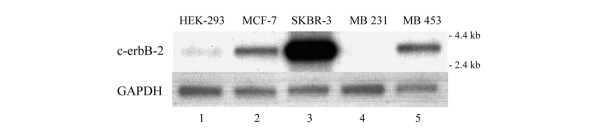
Expression of the *c-erbB-2 *gene in various cell lines. Total RNAs from HEK-293 and human breast cancer cell lines MCF-7, SK-BR-3, MDA-MB-231 and MDA-MB-453 were probed with *c-erbB-2 *cDNA and thereafter with glyceraldehyde 3-phosphate-dehydrogenase (GAPDH) cDNA as a control.

**Figure 2:**
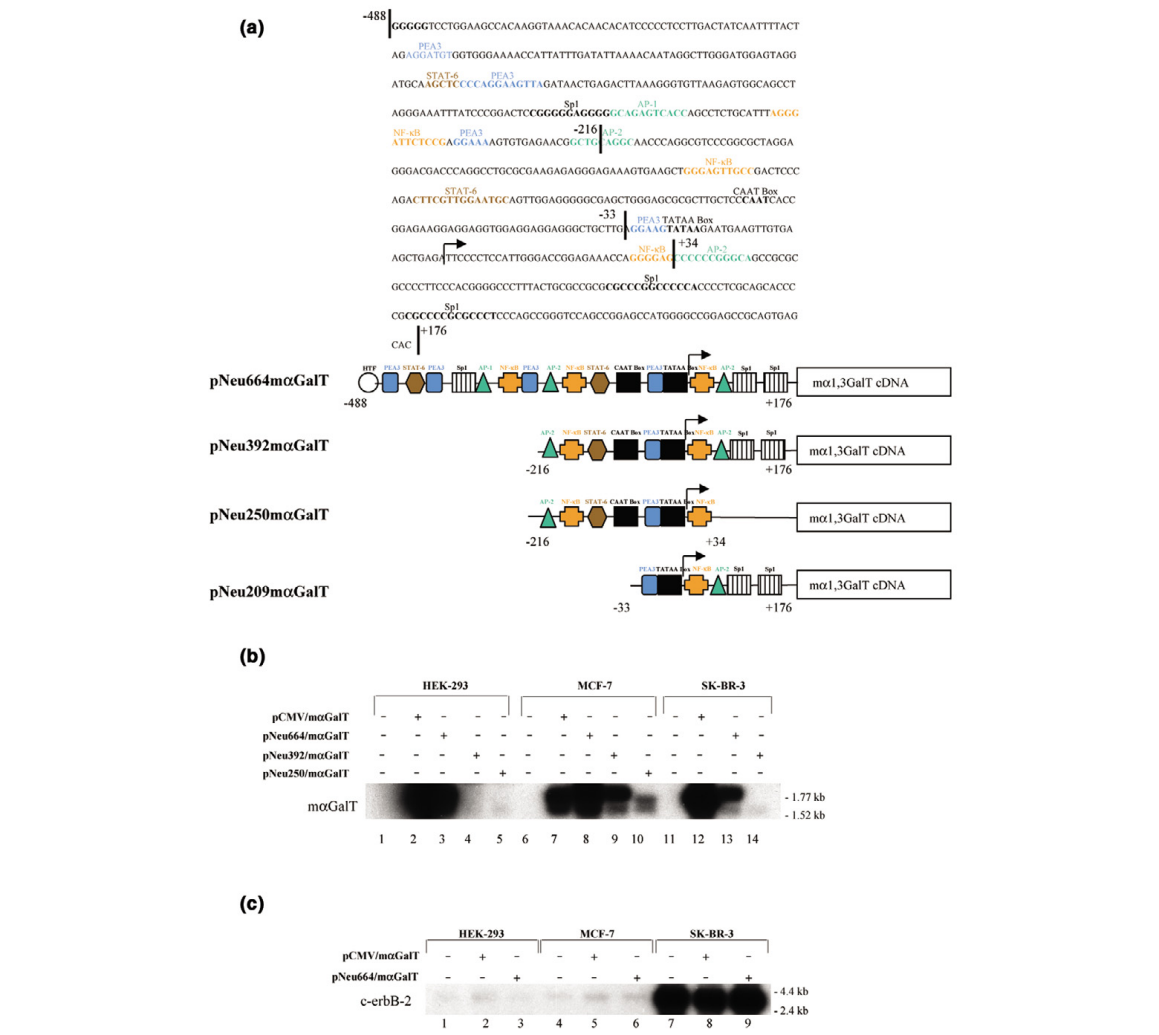
Promoting activity of newly designed forms of p*Neu *in various cell lines differentially expressing *c-erbB-2 ***(a) **Schematic representation of various forms of p*Neu *constructed upstream of the murine α1,3galactosyltransferase (mαGalT) cDNA. The transcription start site is indicated by arrows, and the 5' end points of the various forms of p*Neu *by bold bars. **(b) **Northern blot analysis of expression of mαGalT driven by the CMV promoter (pCMV) and the various forms of constructed p*Neu*. **(c) **Northern blot analysis of *c-erbB-2 *expression in stably transfected HEK-293, MCF-7 and SK-BR-3 cells. Total RNAs were electrophoresed and probed with [α-^32^P]-random-labeled mαGalT or *c-erbB-2 *cDNA, and thereafter with glyceraldehyde 3-phosphate-dehydrogenase (GAPDH) cDNA as a control.

Expression of the endogenous *c-erbB-2 *gene was investigated in various cell lines by northern blotting. c-*erbB-2 *was very weakly expressed in HEK-293 cells and was differentially transcribed in human breast cancer cell lines (Figs 1 and 2C): it was absent in MDA-MB-231, moderately expressed in MCF-7 and MDA-MB-453, and strongly expressed in SK-BR-3 cells. These results are consistent with data published by others [21,30,31]. SK-BR-3 cells are known to overexpress c-erbB-2 as the result of gene amplification in proportions estimated at up to 13:1 [21]. MCF-7 is a known HER2/*neu *non-amplified cell line [20], but various degrees of gene expression have been reported [30,32-34]. The HER2/*neu *transcription level of the MCF-7 cell line used in our laboratory was notably superior to that of the HEK-293 cells (Fig. 1). Serially passaged in different laboratories, MCF-7 cell lines probably exhibit variable levels of HER2/*neu *transcription. Immortalized by adenovirus type 5 (ad5) [35], HEK-293 cells require continuous ad5 E1A expression to proliferate and avoid senescence [36], and can thus be considered subnormal because they are not tumorigenic [37]. Interestingly, their very weak HER2/*neu *expression done them useful as controls to investigate the activation of various forms of p*Neu *as a function of HER2/*neu *expression in breast tumor cells. Because our goal was to take advantage of differential up-regulation of endogenous p*Neu *to overexpress a suicide transgene that is itself driven by an exogenous p*Neu*, MCF-7 cells without HER2/*neu *gene amplification appeared more suitable than HEK-293 cells. SK-BR-3 cells might provide other information, as detailed hereafter.

### Variable activity of mαGalT driven by various forms of *c-erbB-2 *promoter in human breast cancer cell lines

**Figure 3:**
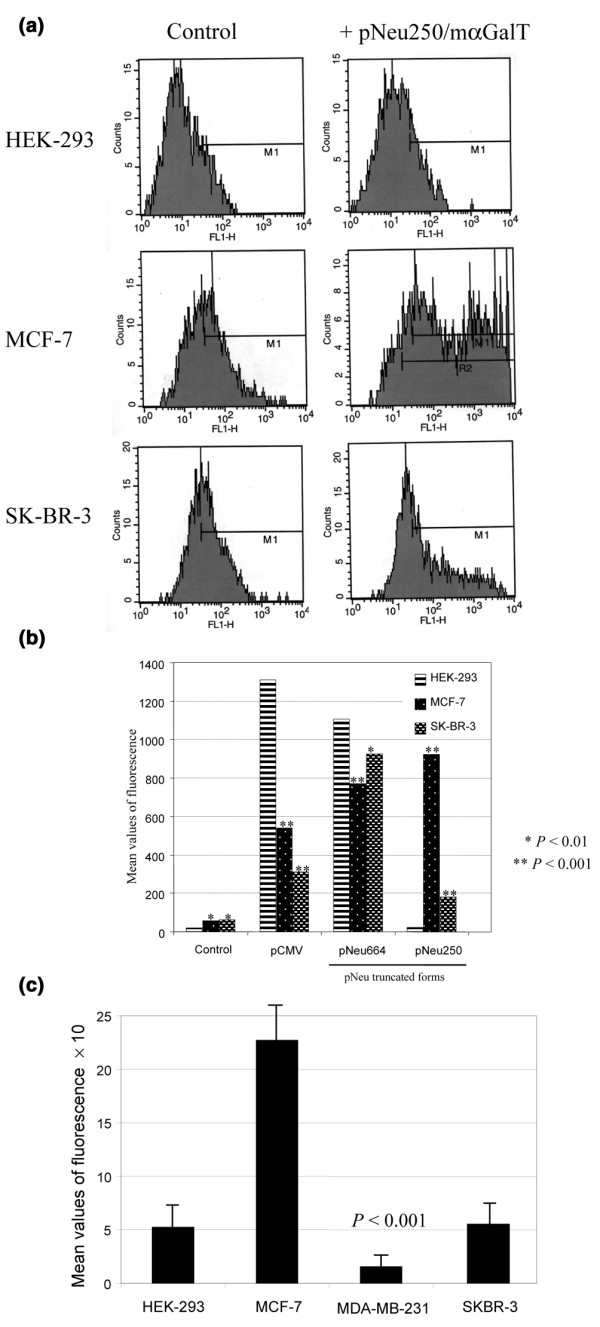
Expression of Galα1,3Gal residues at the surface of transduced cells. Cells stably transduced or not with CMV promoter (pCMV)/murine α1,3galactosyltransferase (mαGalT), p*Neu*664/mαGalT, or p*Neu*250/mαGalT were labeled with fluorescein isothiocyanate-conjugated *Griffonia simplicifolia*/isolectin B4 (FITC-GS-I-B4) lectin (Galα1,3Gal) and analyzed on a FACScan cytometer (counts, cells numbers; FL1-H, fluorescence intensity). **(a) **Binding of FITC-GS-I-B4 to parental and p*Neu*250/mαGalT transduced HEK-293, MCF-7 and SK-BR-3 cells. Mean values of fluorescence detected by flow cytometry at the surface of stably transduced cells with **(b) **the constructs p*Neu*664/mαGalT or p*Neu*250/mαGalT in a plasmid vector (experiments in triplicates), and **(c) **the construct p*Neu*250/mαGalT in a self-inactivating retroviral vector (experiments were conducted using four to five subclones).

Human cells do not express any αGalT activity responsible for αGal epitope expression that is recognized by the GS-I-B4 lectin. GS-I-B4 binding might thus specifically reveal expression of exogenous mαGalT in transgenically modified human cells. A mαGalT cDNA was constructed into pcDNA3 vector downstream of the original pCMV (pCMV/mαGalT). In addition, various forms of p*Neu *were prepared by PCR amplification (Fig. 2a): p*Neu*664 (nucleotides -488/+176, relative to the transcription start site of HER2/*neu*), p*Neu*392 (nucleotides -216/+176) and p*Neu*250 (nucleotides -216/+34). These forms were inserted in the pCMV/mαGalT construct upstream of the mαGalT cDNA, in the place of the original pCMV (p*Neu*664/mαGalT, p*Neu*392/mαGalT and p*Neu*250/mαGalT). These constructs were transferred into HEK-293 control and breast tumor cell lines. Stably transfected cells were analyzed by northern blotting for their expression of αGalT (Fig. 2b) and c-erbB-2 (Fig. 2c), and by flow cytometry for their binding with FITC-GS-I-B4 (Fig. 3a–c). pCMV and p*Neu*664 promoted noticeable expression of αGalT in both HEK-293 and breast cancer cell lines (Fig. 2b, lanes 2–3, 7–8 and 12–13). In contrast, the shortest form, p*Neu*250, raised αGal expression to a more specific level in MCF-7 than in HEK-293 cells (Fig. 2b, compare lane 10 to 5). Similar differential results were observed with p*Neu*392 (Fig. 2b, compare lanes 9 and 14 to 4) whereas a complete switch-off was observed with p*Neu*209 (data not shown). These results were verified phenotypically. Elevated αGal expression was observed in breast tumor and HEK-293 cells with mαGalT driven by pCMV or p*Neu*664, while p*Neu*250 promoted αGal expression only in the breast tumor cells SK-BR-3 and MCF-7 (Fig. 3a). To confirm these results, expression of the cassette p*Neu*250/mαGalT was compared for breast tumor cells expressing and not expressing c-erbB-2. The non-expressing MDA-MB-231 cell line, although appropriate for this purpose, was unfortunately resistant to classic transfection methods. A self-inactivating retroviral vector pseudotyped by a VSV-G glycoprotein was thus used to transfer p*Neu*250/mαGalT. Stably transduced cell lines were subcloned during the course of antibiotic selection. Four to five subclones of each type were analyzed for the expression of mαGalT. The MDA-MB-231 cells, which did not express c-erbB-2, showed a very low level of GS-I-B4 reactivity compared to breast tumor cell lines SK-BR-3 and MCF-7, which express c-erbB-2 (Fig. 3c).

Apparently conflicting results show that p*Neu*250 promoted clearly higher αGal expression in MCF-7 than in SK-BR-3 cells (Fig. 3a,b), while c-erbB-2 was inversely expressed in these two cell lines (Fig. 1, lanes 2 and 3; Fig. 2c, lanes 4 and 7). These data could be explained by the differential mechanisms that sustain HER2/*neu *overexpression, which is regulated at the transcriptional level in MCF-7 cells and is dependent on the existence of multiple gene copies in SK-BR-3 cells. Interestingly, p*Neu*250 promoted a much more specific expression as a function of the differential level of endogenous c-erbB-2 transcription in breast tumor cells (Fig. 1, lanes 2–4) and HEK-293 cells (Fig. 1, lane 1).

### Cytolytic activity of anti-αGal antibodies to transgenically modified breast tumor cells

**Figure 4:**
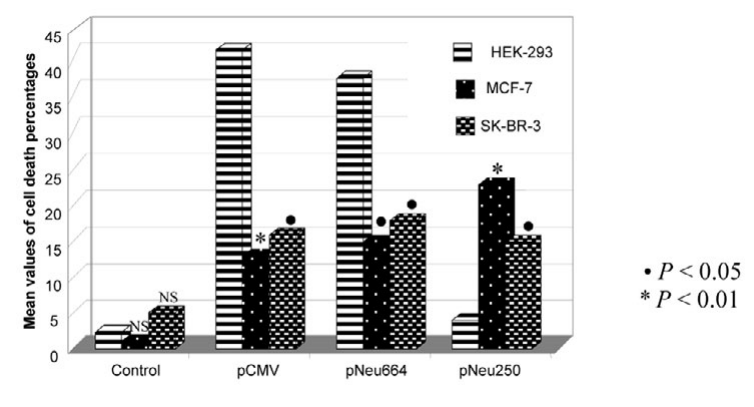
Cytotoxicity assays on HEK-293, MCF-7 and SK-BR-3 cells stably transfected or not with CMV promoter (pCMV)/murine α1,3galactosyltransferase (mαGalT), p*Neu*664/m αGalT, or p*Neu*250/mαGalT. Cells were distributed in 96-well plates (5.10^4^/well) and incubated with human sera, after which rabbit complement was added. Cell death was analyzed using an XTT reagent proliferation assay. Results (means of triplicates) are expressed as percentages of cell death, relative to the amount of formazan formation (absorbance evaluated at 490 nm; NS, not significant).

A high fraction of antibodies from human sera bind αGal epitopes and can efficiently induce the death of cells that exhibit these epitopes via complement activation [4]. Cytotoxicity assays were carried out on HEK-293, MCF-7 and SK-BR-3 cells stably transfected by mαGalT cDNA under the control of p*Neu*664 and p*Neu*250, or pCMV used as a control. The susceptibility of transfected cells to natural human anti-αGal antibodies was verified using a complement-dependent cytotoxicity test (data not shown). Cell death averages were confirmed by an XTT proliferation assay (Fig. 4). All of the cell types transfected with pCMV/mαGalT or p*Neu*664/mαGalT were killed, but to varying degrees (Fig. 4). Interestingly, differential cytolytic activity of antibodies was observed with p*Neu*250/mαGalT, being much greater in MCF-7 and SK-BR-3 cells than in HEK-293 cells (Fig. 4). Here again, mαGalT was much more efficiently driven by pCMV or p*Neu*664 in HEK-293 cells than in breast tumor cells. p*Neu*250 gave differential results that were in favor of the tumor cells; it promoted a more significant proportion of death in MCF-7 than in SK-BR-3 cells (Fig. 4) that correlated with their respective levels of αGal epitope expression (Fig. 3).

## Discussion

Efforts to develop anticancer therapies based on suicide transgenes generally focus on prodrug activating enzymes [38] combined with effective targeting of pathological cells. We have been studying αGalT gene expression and the very high efficiency of natural anti-αGal antibodies in inducing complement-mediated cell killing [39,40] in the field of hyperacute xenograft rejection. We attempted to take advantage of this constitutive immune system to target tumor cells. Several authors have demonstrated the efficacy of natural anti-αGal antibodies for destruction of tumor cells [7-10]. Indeed, the high numbers of circulating oligosaccharides bearing αGal epitopes are responsible for constant booster immunizations. This may explain the high plasma level (1% of IgG) of anti-αGal antibodies in humans [4] and their constant *de novo *synthesis in αGalT knockout mice [41,42]. Natural anti- αGal antibodies are highly cytotoxic and cytolytic as the result of highly efficient complement activation, and this results in hyperacute rejection of xenografts [2,40]. They have also been shown capable of protecting αGalT-deficient mice against engrafted αGal^+ ^colon cancer cells [9].

The purpose of this study was the targeting of tumor cells known to overexpress c-erbB-2 using a selected form of its promoter p*Neu *to drive an active αGalT. This approach was designed to take advantage of the effective antibodies preexisting in all humans. In a similar study, a derived form of the human telomerase promoter was shown to render human pancreatic carcinoma cells susceptible to αGal/complement-mediated cell killing [43]. We selected p*Neu *because it has been well characterized and is overexpressed in a variety of tumors [16,44]. Because definition of a precise p*Neu *sequence with well-restricted activation in tumor cells remains uncertain, however, we analyzed several forms of this promoter. The shortest form, p*Neu*209, which comprises the only PEA3 motif adjacent to the TATA box plus two SP1 sites and one AP-2 site downstream of the transcription start site, promoted very weak αGal expression. The minimal forms, p*Neu*392 and p*Neu*250, were equally capable of selectively inducing αGalT in breast tumor cells compared with HEK-293 cells. We thus concluded that the Ap-2 and SP1 motifs downstream of the transcription start site (Fig. 2) were not essential. The noticeable absence of a CCAAT box in p*Neu*209 probably explains its disrupted activity because in cells over-expressing c-erbB-2, the CCAAT box is up-regulated rather than the TATAA box [45]. Further studies thus focused on comparing p*Neu*250 with the longest form, p*Neu*664. This last form contains several PEA3, NF-*k*B, HER2 transcription factor (HTF) and SP1 sites upstream of the minimal p*Neu*250. The role played by the Ets family and activator protein-2 (AP-2) factors has been extensively studied in breast tumor cells. While the AP-2 binding site was present in both p*Neu*250 and p*Neu*664, the main difference between these forms was the presence of three additional PEA3 motifs in p*Neu*664. Activation of p*Neu*664 was virtually the same in MCF-7 and SK-BR-3 tumor cells, whereas a marked decrease in p*Neu*250 activity was observed only in SK-BR-3 cells (Fig. 3), in complete contrast to their high c-erbB-2 expression (Fig. 2c). As discussed above, the striking overexpression of c-erbB-2 in SK-BR-3 cells can be explained by their multiple gene copies. In MCF-7 cells, the differential promoting activity of p*Neu*250 could be explained by the relative increase in the transcription level compared with HEK-293 cells. In other aspects, in association with *c-erbB-2 *gene amplification, up-regulation of transcriptional factors that control endogenous p*Neu *remains possible. Conflicting results have been published on Ets regulation of c-erbB-2, with activation and repression of p*Neu *by PEA3 factors having been reported [46,47]. The observation that Ets binding leads to a severe bend in DNA could be further support for our findings [46]. When the number of PEA3 binding sites is reduced from four in p*Neu*664 to one in p*Neu*250, over-occupation of the single remaining site in p*Neu*250 might hinder formation of the required DNA conformation rather than favor its reading.

The differential promoting activity of p*Neu*664 and p*Neu*250 in HEK-293 cells (Fig. 3) does not appear to be relevant to the transcriptional regulation of *c-erbB-2 *because this gene is only weakly expressed in these cells (Fig. 1, lane 1). In contrast, HEK-293 cells continuously express ad5 E1A, which has been shown to target p*Neu *[48] as a repressor of HER2/*neu *overexpression [49], and has been proposed for use in cancer gene therapy [50]. Like the Ets factors expressed in tumor cells, an equal level of E1A in HEK-293 cells might activate p*Neu*664, which contains four PEA3 motifs, and repress p*Neu*250, which has only one.

Our efforts to take advantage of natural cytotoxic anti-αGal antibodies as a means of destroying breast tumor cells, and to design a promoter specific for these undesirable cells, have to be considered as a preliminary contribution to the field of cancer gene therapy, given that our results have been obtained in cell line culture models. It has been shown that human primary breast tumors can be successfully engrafted into NOD/SCID mice and maintained in a growing state for more than 100 days [51]. Moreover, αGalT(-/-) KO mice have been fortunately generated by others [52,53]. So to progress towards a gene therapy application, we are developing a two step procedure in mice. First, the distribution and expression of the transgene p*Neu*250/mαGalT, cloned into the retroviral vector pcPMΔU3 (see Materials and methods), will be studied in a human breast cancer xenograft model. Various types of human breast tumor differentially expressing HER2/*neu *will be implanted in NOD/SCID mice, and thereafter the transgene will be injected by a variety of methods. Second, αGalT-transduced tumor pieces will be transplanted into immunocompromised αGalT KO mice to evaluate the tumor destroying activity of purified human anti-αGal antibodies.

## Conclusion

Our results show that the association p*Neu*250/mαGalT could be used to target tumor cells overexpressing c-erbB-2, and thus expose them to the cytolytic activity of natural anti-αGal antibodies. Development of a discriminating *in vivo *system capable of targeting tumor cells according to their level of c-erbB-2 expression could prove beneficial.

## Abbreviations

ad5 = adenovirus type 5; AP-2 = activator protein-2; bp = base pair; FCS = fetal calf serum; FITC-GS-I-B4 = fluorescein isothiocyanate-conjugated *Griffonia simplicifolia*/isolectin B4; αGal = galactose-α1,3galactose-β1,4N-acetylglucosamine-R; αGalT = α1,3galactosyltransferase; GAPDH = glyceraldehyde 3-phosphate-dehydrogenase; LTR = long terminal repeat; mαGalT = murine αGalT; NF-*k*B = nuclear factor *k*B; pCMV = CMV promoter; PCR = polymerase chain reaction; p*Neu *= HER2/*neu *promoter; VSV-G = vesicular stomatitis viral G glycoprotein.

## Competing interests

The author(s) declare that they have no competing interests.

## Authors' contributions

ML performed the various p*Neu *constructs, transduction assays, Northern blot analyses and participated in literature search and critical reading of the manuscript. LO conducted the statistical analysis and participated in cell proliferation assays. VG carried out flow cytometry analysis and contributed in Northern blot analyses. JCL conceived the study and further developments, looked after data interpretation, and wrote the manuscript. All authors read and approved the final manuscript.
